# Long‐term efficacy of 10 kHz spinal cord stimulation in managing painful diabetic neuropathy: A post‐study survey

**DOI:** 10.1111/papr.70023

**Published:** 2025-04-17

**Authors:** Erika A. Petersen, Shawn M. Sills, Thomas G. Stauss, Rose Province‐Azalde, Michael J. Jaasma, Deborah R. Edgar, Judith L. White, James A. Scowcroft, Cong Yu, Jijun Xu, Maged N. Guirguis, Kasra Amirdelfan, David J. DiBenedetto, Ali Nairizi, Shivanand P. Lad, Neel D. Mehta, Dawood Sayed, Khalid A. Sethi, Sarah Benducci, Manish Bharara

**Affiliations:** ^1^ University of Arkansas for Medical Sciences Little Rock Arkansas USA; ^2^ Touchstone Interventional Pain Center Medford Oregon USA; ^3^ Advanced Pain Management Greenfield Wisconsin USA; ^4^ Nevro Corp Redwood City California USA; ^5^ Commexus Ltd Stirling UK; ^6^ AES Compass Orlando Orlando Florida USA; ^7^ Pain Management Associates Lee's Summit Missouri USA; ^8^ Swedish Medical Center Seattle Washington USA; ^9^ Department of Anesthesiology and Pain Management Cleveland Clinic Cleveland Ohio USA; ^10^ Ochsner Health New Orleans Louisiana USA; ^11^ IPM Medical Group Walnut Creek California USA; ^12^ Boston PainCare Waltham Massachusetts USA; ^13^ Nevada Advanced Pain Specialists Reno Nevada USA; ^14^ Department of Neurosurgery Duke University Durham North Carolina USA; ^15^ Department of Anesthesiology Weill Cornell Medicine New York New York USA; ^16^ Department of Anesthesiology The University of Kansas Kansas City Kansas USA; ^17^ Department of Neurosurgery United Health Services Johnson City New York USA

## Abstract

**Objective:**

To evaluate the longer‐term efficacy of 10 kHz spinal cord stimulation (SCS) in managing painful diabetic neuropathy (PDN) in a routine clinical setting after the transition from the 24‐month SENZA‐PDN study.

**Methods:**

We contacted 142 participants who completed 24 months of postimplantation follow‐up in the former randomized controlled trial (SENZA‐PDN). Of these, 57 consented and responded to this longer‐term post‐study survey. Outcomes assessed included pain relief, health‐related quality of life (HRQoL) measured using the EuroQol 5‐Dimensional 5‐Level (EQ‐5D‐5L) instrument, Patient Global Impression of Change (PGIC), HbA1c, and weight.

**Results:**

Our survey captured patient‐reported outcomes at a median of 4.1 years after implantation of a permanent 10 kHz SCS system. Among the surveyed participants, 76.8% (43 of 56) reported clinically meaningful pain relief (≥2 points), and 84.6% (44 of 52) achieved a clinically meaningful improvement in their EQ‐5D‐5L index score, with a final mean EQ‐5D‐5L index score of 0.825. Additionally, 74.5% (38 of 51) reported being “Better” or “A great deal better” on the PGIC scale. The surveyed participants reported a mean HbA1c level decrease of 0.4% (*p* = 0.027), with a more substantial improvement of 1.6% (*p* < 0.001) among those with type 2 diabetes (T2D) and a higher preimplantation HbA1c (>8%). Significant weight loss was also observed, with a mean reduction of 7.0 kg (*p* < 0.001) in the overall cohort and 8.7 kg (*p* < 0.001) in the subgroup with T2D and a higher BMI at preimplantation (≥35 kg/m^2^).

**Conclusions:**

High‐frequency SCS at 10 kHz provided sustained and clinically meaningful improvements in pain and HRQoL for PDN patients at 4.1 years postimplantation, with no explants in the cohort due to inefficacy. Alongside these benefits, participants experienced metabolic changes that included reductions in body weight and HbA1c beyond that achieved at 24 months, although changes in lifestyle and medication were not accounted for in this analysis. Notably, the cohort's final mean EQ‐5D‐5L index score was comparable to the US norm. These findings support 10 kHz SCS as a durable and effective treatment option for PDN in routine clinical practice.

## INTRODUCTION

Diabetic neuropathy (DN) affects approximately 50% of individuals with diabetes.^1^ The most prevalent form of DN is distal symmetrical length‐dependent polyneuropathy,^1^ which typically manifests with sensory issues in the early stages.^2^, ^3^ Among the more challenging complications associated with DN, painful diabetic neuropathy (PDN) is particularly debilitating and affects up to 25% of all people with diabetes.^4^ PDN is characterized by diverse and severe pain symptoms, including burning, shooting or stabbing pains, tingling, and electric shock‐like sensations.^2^ As with sensory issues, these pain symptoms most commonly start in the feet, progressing proximally into the lower limbs, with involvement of the hands and upper limbs in the advanced stages.^2^, ^3^ Symptoms negatively impact sleep, mood, and daily functioning, collectively leading to poor health‐related quality of life (HRQoL).^5^, ^6^, ^7^, ^8^ Damage to peripheral nerve autonomic fibers can also lead to symptoms such as abnormal sweating, dry skin, bladder dysfunction, gastrointestinal issues, and sexual dysfunction.^9^, ^10^ Moreover, some research shows that PDN is associated with higher body mass index and poorer glycemic control compared to painless DN, thereby further complicating diabetes management and prognosis.^11^, ^12^, ^13^

Current medical guidelines recommend several oral pharmacological interventions for the treatment of PDN pain symptoms, including anticonvulsants (eg, pregabalin, gabapentin), selective serotonin reuptake inhibitors (eg, duloxetine), and tricyclic antidepressants (eg, amitriptyline).^2^ However, these medications often fail to provide adequate pain relief and are associated with side effects that limit long‐term adherence.^14^, ^15^, ^16^, ^17^, ^18^, ^19^, ^20^, ^21^ Furthermore, the evidence base underpinning these treatments is mainly derived from studies with short follow‐up durations, leaving uncertainty regarding their long‐term efficacy.

Spinal cord stimulation (SCS) is a well‐established alternative therapeutic option for managing chronic neuropathic pain.^22^, ^23^, ^24^, ^25^ The SCS technique involves the insertion of electrodes into the epidural space to deliver electrical pulses directly to the spinal cord. Conventional low‐frequency SCS (LF‐SCS) at 40–60 Hz masks pain by inducing paresthesia over the affected area. In contrast, high‐frequency SCS (≥1 kHz) can relieve pain without the persistent feeling of paresthesia, which patients may dislike. Only one paresthesia‐free SCS modality, 10 kHz SCS, has been evaluated in the PDN indication. In a long‐term randomized control trial (SENZA‐PDN), the therapy demonstrated remarkable outcomes over 24 months, with 10 kHz SCS recipients showing a significant reduction in lower limb pain (80%), as well as improvements in sleep quality, neurological function, and protective sensation.^26^, ^27^ Additional benefits included improved HRQoL and reduced glycated hemoglobin (HbA1c) levels and weight.^28^, ^29^

Related research in patients with PDN has suggested that 10 kHz SCS may have a pro‐regenerative effect on peripheral nerve fibers. Specifically, Chen et al. demonstrated significantly increased intraepidermal nerve fiber density (IENFD) in the proximal thigh and distal legs in PDN patients treated with 10 kHz SCS over 12 months in a small feasibility study.^30^ Moreover, Kissoon et al. observed a beneficial effect of the therapy on autonomic symptoms in patients with PDN, with a significant reduction in sweat volumes in the proximal leg after 12 months of treatment.^31^ Both studies also found improvements in sensory function at the 12‐month mark.

While the SENZA‐PDN study has provided robust evidence supporting the efficacy of 10 kHz SCS in managing PDN over 24 months,^26^ the longer‐term durability of this therapy in routine clinical settings remains to be established. The present study aims to address this gap by evaluating the benefits of 10 kHz SCS in participants from the SENZA‐PDN study after transitioning back to routine clinical care, thereby assessing the sustainability of the therapy in a real‐world healthcare environment.

## METHODS

### SENZA‐PDN study overview

Previous publications have comprehensively reported details of the SENZA‐PDN study design and results.^26^, ^32^, ^33^, ^34^ Briefly, we enrolled patients from 18 centers in the US who had experienced PDN symptoms for at least 12 months without relief from standard pharmacological neuropathic pain treatments. The study protocol was approved by a central institutional review board (IRB; Western IRB: #20171535) and local site IRBs as necessary. All participants provided written informed consent.

Recruitment criteria required participants to have lower limb pain ≥5 cm on a 10‐cm visual analog scale (VAS), HbA1c ≤10% (86 mmol/mol), body mass index (BMI) ≤45 kg/m^2^, and daily opioid usage ≤120 mg morphine equivalents. Participants were randomly assigned to receive either conventional medical management (CMM) alone for PDN or 10 kHz SCS in addition to CMM. After 6 months, the study protocol permitted optional crossover to the alternative treatment arm if pain relief was inadequate.

Before implanting a permanent 10 kHz SCS system, participants underwent a temporary stimulation trial for 5–7 days to assess their response. During this trial, stimulation leads were placed percutaneously into the epidural space under fluoroscopic guidance and connected to an external pulse generator. If the trial was successful, defined as pain relief of at least 50%, the participant was eligible to receive a permanent 10 kHz SCS system. After permanent implantation, a therapy consultant employed by the device manufacturer (Nevro Corp.) individually optimized stimulation parameters to ensure maximum pain relief. Throughout the SENZA‐PDN study, all participants continued to receive CMM as required. Individuals who received a permanent 10 kHz SCS system were followed for 24 months postimplantation.

During the study, participants recorded their lower limb pain intensity on a 0‐10‐point VAS.^35^ They also rated their health‐related quality of life using the EuroQol 5‐Dimensional 5‐Level instrument (EQ‐5D‐5L),^36^ a validated patient‐reported outcome measure that assesses 5 dimensions of health: mobility, self‐care, usual activities, pain/discomfort, and anxiety/depression. Additionally, participants rated their overall change in health status using the 7‐point Patient Global Impression of Change (PGIC) scale, from “no change (or condition has got worse)” to “a great deal better.” Furthermore, standard laboratory blood tests were conducted to determine HbA1c levels, and weight measurements were recorded.

In total, 142 individuals from the SENZA‐PDN study—including 84 original and 58 crossover participants—received a permanent 10 kHz SCS system and completed the final 24‐month postimplantation assessment.^26^

### Post‐study survey

Participants returned to routine clinical care after completing and exiting the SENZA‐PDN study. If required, Nevro therapy consultants remain available to adjust and optimize stimulation parameters as part of standard SCS industry practice during routine clinical care. Approximately 2 years after the completion of the SENZA‐PDN study, a Nevro therapy consultant attempted to contact the former study participants for this single‐instance post‐study survey, with a minimum of 3 attempts before considering them a survey “non‐completer”. Notably, under an IRB‐approved protocol (IRB# 20210346), patients were consented for the post‐study survey upon first contact. Following consent, the verbal survey asked participants to report their current lower limb pain intensity on a 0–10‐point numerical rating scale (NRS) with “no pain” being the lowest and “worst pain imaginable” as the highest score. While the clinical setting of the original SENZA‐PDN study allowed the use of a VAS pain scale, we used the NRS pain scale for the post‐study survey to accommodate telephone‐based data collection.^37^ Lastly, they completed the EQ‐5D‐5L and PGIC questionnaires, and provided their most recent HbA1c level and weight measurement (both within the last 6 months).

### Statistical analysis

Statistical analyses were performed using SAS (Version 9.4, SAS Institute Inc., Cary, NC). Using data from the SENZA‐PDN study,^26^ we evaluated outcomes at preimplantation and 24 months postimplantation for completers of the post‐study survey. We also assessed longer‐term post‐study survey outcomes in the same cohort.

The analysis of time and effects for continuous and ordinal variables used a repeated‐measures mixed‐model ANOVA with Tukey pairwise comparisons. The repeated‐measures model included time as a fixed effect and participant as a random effect. We used a two‐independent sample *t*‐test to compare continuous outcomes between completers and non‐completers of the longer‐term survey and a chi‐square test to compare categorical outcomes. Results for the preimplantation and 24‐month visits include only SENZA‐PDN study participants who completed the post‐study survey. For the post‐study survey, results are presented for all available data.

In order to investigate if a bias was created due to differences between those who responded to the survey versus those who did not, a 2‐sample *t*‐test was performed to compare the 24‐month pain, HbA1c, weight, and EQ‐5D‐5L outcomes between the two groups. A chi‐square test was used to compare 24‐month PGIC responses between the groups.

In addition to evaluating mean pain scores and the reduction in mean pain from preimplantation at the 24‐month and longer‐term survey, we also determined the proportion of participants who experienced clinically meaningful pain relief, defined as at least a 2‐point or 30% reduction in pain score from preimplantation, in line with the established minimally important clinical difference (MCID) for pain relief in patients with chronic pain.^38^ Similarly, we evaluated mean EQ‐5D‐5L index scores, the mean change in index score from preimplantation at the 24‐month and post‐study survey, and the proportion of participants whose index score improved by at least 0.03, based on the MCID of 0.03–0.05 for adults with type 2 diabetes.^39^

## RESULTS

### Study population

Approximately 2 years after the end of the SENZA‐PDN study, 57 former study participants, with a mean age of 62.7 years (range: 36–81), completed the post‐study survey and were included in the present analysis. During the median (IQR) time of 4.1 (0.51) years since implantation, no devices were explanted in this cohort because of loss of efficacy. Among the 85 survey non‐completers, 83 were either unreachable or declined to participate, and two had died from causes unrelated to the device. A comparison of outcome measures at the final 24‐month SENZA‐PDN study visit, including pain relief, EQ‐5D‐5L, PGIC, HbA1c, and weight, found no statistical differences between survey completers and non‐completers (Table S1).

### Pain relief

**FIGURE 1:**
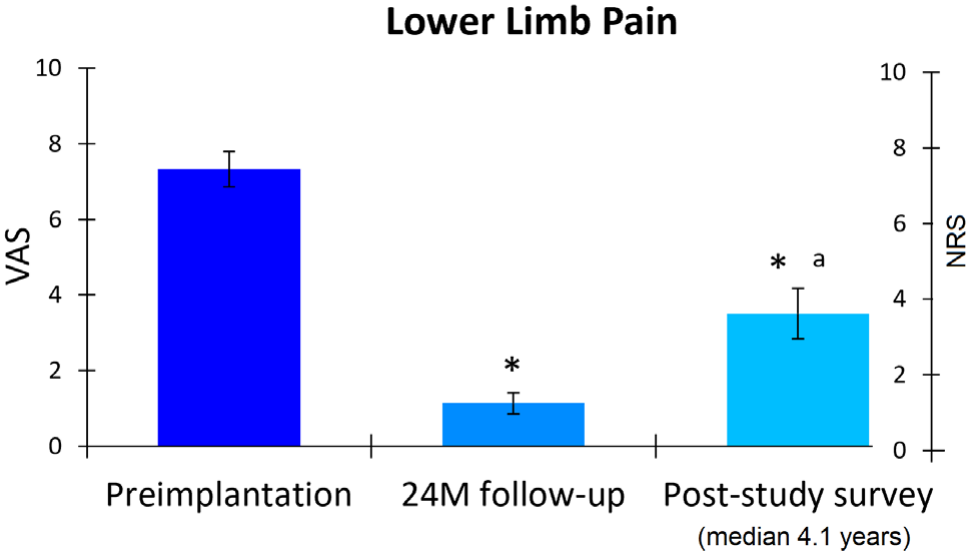
Lower limb pain on a 0–10‐point visual analog scale (VAS) at preimplantation, 24 months (24 M) postimplantation, and 0–10‐point numerical rating scale (NRS) scale at the post‐study survey (median of 4.1 years postimplantation) in the post‐study survey completers. Error bars indicate 95% CI; *p < 0.001 versus preimplantation; ^a^ *p* < 0.001 versus 24 M follow‐up.

Participants reported lower limb pain intensity using either a visual analog scale (VAS; 0–10 cm; SENZA‐PDN study) or a numerical rating scale (NRS; 0–10 points; post‐study survey). At preimplantation, the cohort had moderate‐to‐severe neuropathic pain, indicated by a mean VAS score of 7.3 cm (95% CI, 6.9–7.8; Figure 1). After 24 months of treatment, pain intensity significantly reduced by 6.2 points (95% CI, 5.7–6.7; *p* < 0.001), with the mean VAS score decreasing to 1.1 cm (95% CI, 0.9–1.4). Notably, all participants (57 of 57) achieved a clinically meaningful ≥2‐point improvement in pain at 24 months. In the longer‐term survey, participants reported a mean NRS score of 3.5 points (95% CI, 2.8–4.2), representing a significant mean reduction of 52.7% or 3.9 points (95% CI, 3.1–4.7; *p* < 0.001) from preimplantation and a small significant increase from 24 months (*p* < 0.001). Additionally, 76.8% (43 of 56) of the cohort maintained a clinically meaningful improvement in pain at the post‐study survey.

### Health‐related quality of life (HRQoL)

**FIGURE 2:**
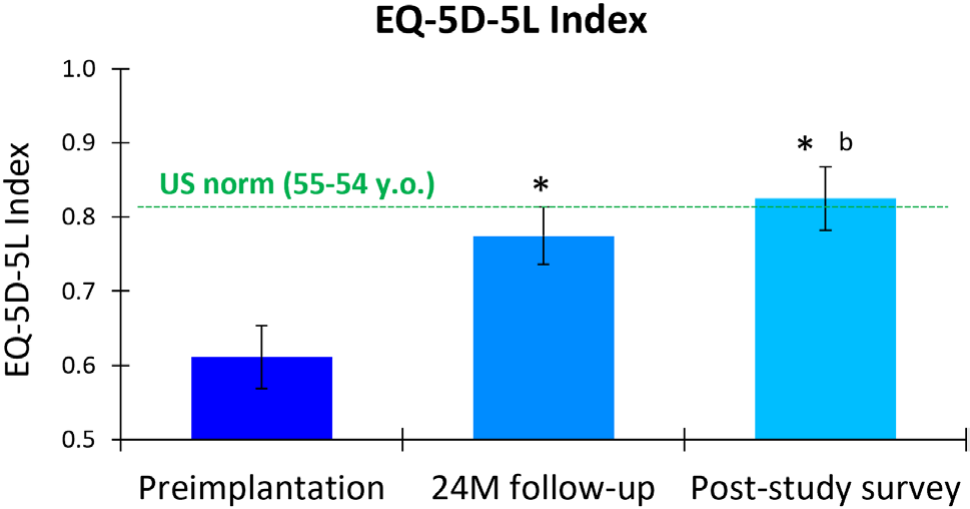
The EuroQol 5‐Dimensional 5‐Level (EQ‐5D‐5L) index score in the post‐study survey completers at preimplantation, 24 months (24 M) postimplantation, and at the post‐study survey (mean of 4.1 years postimplantation). The dotted line indicates the US norm of 0.815 for the age group of 55–64 years.^35^ Error bars indicate 95% CI; *p < 0.001 versus preimplantation; ^b^ *p* < 0.024 versus 24 M follow‐up.

The EuroQol 5‐Dimensional 5‐Level (EQ‐5D‐5L) questionnaire revealed significant improvements in participants' health‐related quality of life (HRQoL) after treatment with 10 kHz SCS. The mean index score increased from 0.611 (95% CI, 0.569–0.653) at preimplantation to 0.775 (95% CI, 0.736–0.813) at 24 months, a significant mean increase of 0.164 (95% CI, 0.122–0.205; *p* < 0.001; Figure 2). The longer‐term survey showed a further improvement in mean score to 0.825 (95% CI, 0.782–0.868), representing a significant mean improvement of 0.227 (95% CI, 0.181–0.273; *p* < 0.001) from preimplantation and a significant mean increase from the 24‐month score (*p* = 0.024). Using an MCID threshold of 0.03 for adults with type 2 diabetes,^39^ 80.7% (46 of 57) and 84.6% (44 of 52) of the cohort experienced clinically meaningful HRQoL improvements at 24 months and the longer‐term survey, respectively, compared to preimplantation.

### Patient global impression of change (PGIC)

**FIGURE 3:**
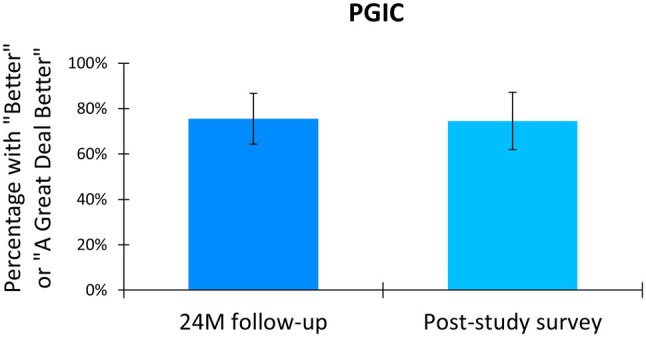
Patient Global Impression Change (PGIC) scale at preimplantation, 24 months (24 M) postimplantation, and at the post‐study survey (mean of 4.1 years postimplantation) in the post‐study survey completers.

Participants reported their perceived change in health status after treatment with 10 kHz SCS using the Patient Global Impression of Change (PGIC) scale. After 24 months of treatment, 75.4% (43 of 57) of the cohort reported feeling “better” or “a great deal better” than their status before implantation (Figure 3). This proportion was sustained at the longer‐term survey, with 74.5% (38 of 51) of the cohort reporting health status improvement.

### Glycemic control

**FIGURE 4:**
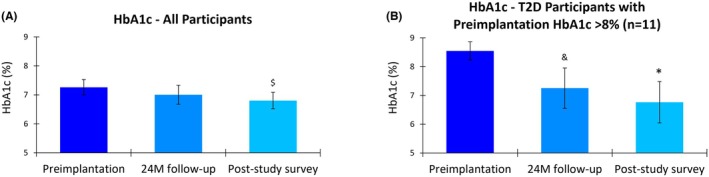
Glycated hemoglobin (HbA1c) level (%) at preimplantation, 24 months (24 M) postimplantation, and at the post‐study survey (mean of 4.1 years postimplantation) in (A) the post‐study survey completers and (B) the subgroup of post‐study survey completers with type 2 diabetes (T2D) and preimplantation HbA1c >8%. Error bars indicate 95% CI; ^$^ *p* = 0.027 versus preimplantation; ^&^ *p* = 0.009 versus preimplantation; *p < 0.001 versus preimplantation.

Prior to implantation, participants exhibited suboptimal glycemic control, indicated by a mean HbA1c of 7.3% (95% CI, 7.0–7.5). After 10 kHz SCS therapy, the mean HbA1C slightly decreased to 7.0% (95% CI, 6.7–7.3) at 24 months and further improved to 6.8% (95% CI, 6.5–7.1) at the longer‐term survey (Figure 4). Although the mean change in HbA1c between preimplantation and 24 months did not reach statistical significance (*p* = 0.153), the mean reduction of 0.4% (95% CI, 0.1–0.8) from preimplantation to the longer‐term survey was statistically significant (*p* = 0.027).

A subgroup analysis of those with type 2 diabetes (T2D) and a preimplantation HbA1c >8% (*n* = 11) demonstrated more pronounced improvements in glycemic control over time. In this subgroup, the mean HbA1c decreased from 8.5% (95% CI, 8.2–8.9) at preimplantation to 7.3% (95% CI, 6.5–8.0) at 24 months, a statistically significant mean reduction of 1.1% (95% CI, 0.6–1.7; *p* = 0.009). In the longer‐term survey, the mean HbA1c further decreased to 6.8% (95% CI, 6.0–7.5), representing a statistically significant mean reduction of 1.6% (95% CI, 1.0–2.3; *p* < 0.001) compared to preimplantation in the smaller subgroup.

### Weight

A progressive and significant reduction in mean weight was observed in the study cohort over the evaluation period. The mean weight decreased from 106.2 kg (95% CI, 101.2–111.2) at preimplantation to 102.4 kg (95% CI, 97.1–107.6) at 24 months, and further reduced to 99.7 kg (95% CI, 94.5–105.0) at the longer‐term survey. The mean reduction in weight of 3.3 kg (95% CI, 0.9–5.8) from preimplantation to 24 months was statistically significant (*p* = 0.032), as was the mean decrease of 7.0 kg (95% CI, 4.3–9.7) from preimplantation to the longer‐term survey (*p* < 0.001), with significant additional weight loss between 24 months and the longer‐term survey (*p* = 0.017).

**FIGURE 5:**
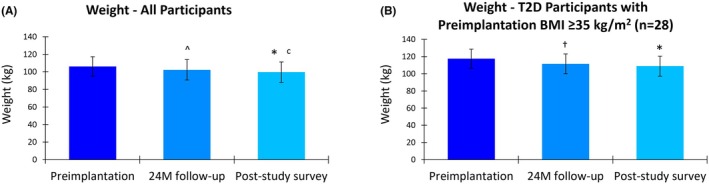
Weight (kg) at preimplantation, 24 months (24 M) postimplantation, and at the post‐study survey (mean of 4.1 years postimplantation) in (A) the post‐study survey completers and (B) the subgroup of post‐study survey completers with type 2 diabetes (T2D) and preimplantation body mass index (BMI) ≥35 kg/m^2^. Error bars indicate 95% CI; ^*p* = 0.032 versus preimplantation; *p < 0.001 versus preimplantation; ^c^ *p* < 0.017 versus 24 M follow‐up; ^†^ *p* = 0.026 versus preimplantation.

Participants with T2D and a BMI ≥35 kg/m^2^ at preimplantation (*n* = 28) also experienced significant weight loss. In this subgroup, the mean weight prior to implantation was 117.7 kg (95% CI, 112.7–122.6), which reduced to 111.6 kg (95% CI, 106.5–116.7) after 24 months of 10 kHz SCS therapy (Figure 5). The mean decrease in weight of 4.9 kg (95% CI, 1.1–8.8) was statistically significant (*p* = 0.026). In the longer‐term survey, the mean weight in this subgroup further decreased to 108.9 kg (95% CI, 103.1–114.8), a statistically significant mean reduction of 8.7 kg (95% CI, 5.1–12.4; *p* < 0.001) from preimplantation.

## DISCUSSION

This longer‐term post‐study survey demonstrated the sustained efficacy of 10 kHz SCS therapy in managing painful diabetic neuropathy (PDN) during routine clinical care. Conducted approximately 2 years after the completion of the SENZA‐PDN study, our survey evaluated participants at a median of 4.1 years postimplantation. Our longer‐term results showed that survey completers continued to experience significant and clinically meaningful improvements in pain and health‐related quality of life (HRQoL), glycemic control, and weight, even after returning to routine clinical care, with 75% of surveyed participants reporting improved health status compared to preimplantation.

The durable impact of 10 kHz SCS on pain in our survey completers was evident, with 77% of participants reporting clinically meaningful pain relief during the longer‐term survey despite the refractory nature of PDN pain symptoms. Moreover, the EQ‐5D‐5L index score, which measures health‐related quality of life (HRQoL), showed substantial improvement, highlighting the multidimensional benefits of 10 kHz SCS in this population. The mean gain in an index score of 0.227 at the longer‐term survey was both statistically significant and clinically important (ie, 7.5 times the MCID of 0.03 in T2D),^39^ with a significant increase in mean index score between 24 months and the longer‐term survey.

Notably, the final mean EQ‐5D‐5L index score of 0.825 in our study cohort exceeds the reported US population norm of 0.815 for the age group of 55–64 years.^40^ Additionally, 52% of participants (27 of 52) with available data had a final index score at or above 0.815, indicating a restoration of HRQoL consistent with the general population in the 55–64 age group. These findings suggest that 10 kHz SCS has a durable and cumulative effect on overall well‐being. In contrast, patients treated with LF‐SCS therapy showed no significant improvement in baseline EQ‐5D‐5L index score after 2 years of treatment,^41^ consistent with longer‐term data published after 8–10 years of treatment for 19 patients that showed maintained pain relief, but also found no improvement in index score.^42^

In addition to the clinically important improvements in pain and HRQoL, our results suggest that 10 kHz SCS therapy may offer beneficial secondary effects on glycemic control and weight. In our study cohort, the HbA1c level was significantly reduced by a mean of 0.4% at the longer‐term survey. Notably, the subgroup with T2D and a higher preimplantation HbA1c level (>8%) experienced a more substantial mean decrease in HbA1c of 1.6% at the longer‐term survey. This improvement surpasses the 0.5% threshold considered clinically meaningful.^43^, ^44^ In parallel with improvements in HbA1c, the study cohort also demonstrated progressive and significant weight loss, with a final mean reduction in weight of 7.0 kg, or 6.6% of the preimplantation weight. In the subgroup with T2D and a higher BMI at preimplantation (≥35 kg/m^2^), weight loss was similar in percentage terms at the longer‐term survey, with a significant mean reduction of 8.7 kg, or 7.4%. Although medication changes and lifestyle modifications may have influenced these improvements in HbA1c and weight, the reduction in pain could have facilitated better diabetes self‐management behaviors and medication adherence, a prospect that warrants further investigation in a larger cohort. In addition, there is some evidence of a mechanism of action particular to 10 kHz SCS that could have a more direct effect on HbA1c and weight, such as modulation of autonomic tone.^31^, ^45^, ^46^

The improvements seen in non‐pain outcomes in this longer‐term survey highlight the importance of considering therapy benefits beyond pain relief alone. These findings align with the Initiative on Methods, Measurement, and Pain Assessment in Clinical Trials (IMMPACT) survey of people with chronic pain, which emphasizes that patients value additional core outcomes from chronic pain therapy other than pain relief, including physical functioning and emotional well‐being.^47^ A recent study by Heros et al.^48^ further supports this holistic approach to assessing SCS therapy outcomes. Their analysis found that the PGIC instrument may better predict long‐term treatment efficacy and patient satisfaction than the NRS pain scale.

The present study has several strengths, including its average follow‐up duration of over 4 years and routine clinical setting. However, some limitations should be acknowledged. First, the longer‐term data represent a subset of the original SENZA‐PDN study population because not all SENZA‐PDN study participants responded to the survey request. Still, at the end of the original study, the patients who responded to the survey were not significantly different from those for whom longer‐term data could not be collected, suggesting there was not a significant bias based on survey response.

It is important to note that we assessed pain scores using different scales during the SENZA‐PDN study (VAS) and at the longer‐term follow‐up (NRS). While VAS and NRS are often used interchangeably and analyzed as equivalent data, they do not always agree.^49^ It is possible that patients may rate their pain differently on a discrete scale (NRS) compared to a more nuanced continuous scale (VAS). Additionally, fluctuations in pain severity,^50^ changes in medication regimens, and the transition from a controlled study environment to routine clinical care could have influenced patients' pain perception and/or adherence to pain management strategies. In the post‐study survey, HbA1c and weight measurements were based on patient reporting, which could be subject to recall errors. Our analysis did not account for potential changes in lifestyle or diabetes management medications since the completion of the original study. Consequently, the reductions in HbA1c and body weight may have resulted from lifestyle modifications (eg, diet and physical activity), adjustments in medication doses, and/or changes to medications. Finally, missing data may have impacted the results. Therefore, the results from this analysis should be interpreted with caution.

## CONCLUSIONS

This longer‐term post‐study survey, at a mean duration of 4.1 years postimplantation, demonstrates the long‐term durability of 10 kHz SCS in managing PDN. Patients continued to experience clinically meaningful pain relief, significant improvements in HRQoL, and sustained benefits in overall health status, as reflected by the PGIC results. Reductions in weight and HbA1c were also maintained, although changes in lifestyle and medication were not accounted for in this analysis. In addition, this cohort had no explants due to inefficacy during the follow‐up period. The observed improvements in multiple areas of patient overall health support the use of 10 kHz SCS as a durable, effective, and holistic treatment option for PDN in routine clinical practice.

## AUTHOR CONTRIBUTIONS

Study conception and design: E. Petersen, R. Province‐Azalde, S. Benducci, M. Bharara; data collection: E. Petersen, S. Sills, T. Stauss, J. White, J. Scowcroft, C. Yu, J. Xu, M. Guiguis, K. Amirdelfan, D. DiBenedetto, A. Nairizi, S. Lad, N. Mehta, D. Sayed, K. Sethi; analysis and interpretation of results: E. Petersen, M. Jaasma, D. Edgar, S. Benducci, M. Bharara, R. Province‐Azalde. All authors reviewed the results and approved the final version of the manuscript.

## FUNDING INFORMATION

This study was funded by Nevro Corp.

## CONFLICT OF INTEREST STATEMENT

Erika A. Petersen has received consulting fees from Abbott Laboratories, Biotronik, Boston Scientific, Medtronic Neuromodulation, Nalu Medical, Neuros Medical, Nevro Corp, Presidio Medical, Saluda, and Vertos Medical, research support from Mainstay, Medtronic Neuromodulation, Nalu Medical, Neuros Medical, Nevro Corp, ReNeuron, Saluda, and SPR, and stock options from neuro42 and SynerFuse. Shawn M. Sills has received research support from Nevro Corp. Thomas G. Stauss has received research support from Nevro Corp. Rose Province‐Azalde, Michael J. Jaasma, Sarah Benducci, and Manish Bharara were employees of Nevro Corp during work on study and manuscript. Deborah R. Edgar received a fee from Nevro Corp. for the preparation of this manuscript in her capacity as an independent medical writer. Judith L. White has received consulting fees from California Institute for Biomedical Research and Eli Lilly and research support from Nevro Corp. James A. Scowcroft has received research support from Boston Scientific, Nevro Corp, Saluda Medical, and Vertiflex. Cong Yu has received research support from Nevro Corp. Jijun Xu has received research support from the Cleveland Clinic Velosano Program, the National Institutes of Health, the Steve and Melody Golding Foundation, and Nevro Corp. Maged N. Guirguis has received consulting fees from Abbott Laboratories, Avanos Medical, Avertis Pharmacy, Boston Scientific, Nevro Corp, and Saluda Medical, as well as research support from Abbott Laboratories, Avanos Medical, Boston Scientific, Nalu Medical, Neuros Medical, Nevro Corp, and Saluda Medical. Kasra Amirdelfan has received consulting fees from Biotronik, Medtronic, Nalu Medical, Nevro Corp, and Saluda Medical, as well as research support from Biotronik, IPM Medical Group, Nevro Corp, Saluda Medical, SPR Therapeutics, and Vivex Biologics. David J. DiBenedetto has received research support from Nevro Corp, as well as funding for serving as principal investigator of a study supported by SPR Therapeutics paid to his institution. Ali Nairizi has received consulting fees from Aurora Spine, Flowonix, and Nevro Corp as well as research support from Nevro Corp. Shivanand P. Lad has received consulting fees from Nevro Corp and research support from Nevro Corp. Neel D. Mehta has received consulting fees from Averitas, Nevro Corp, and Salix Pharmaceuticals, as well as research support from Boston Scientific and Nevro Corp. Dawood Sayed has received consulting fees from Abbott Laboratories, Boston Scientific, Flowonix, Medtronic, Nevro Corp, Vertiflex, and Vertos Medical, as well as research support from Abbott Laboratories, Biotronic, Nevro Corp, Vertiflex, and Vertos Medical. Khalid A. Sethi has received research support from Nevro Corp.

## INFORMED CONSENT

All participants provided informed consent.

## IRB APPROVAL STATEMENT

The study protocol was approved by a central institutional review board (IRB; Western IRB: #20210346, 02/27/2024).

## Supporting information


Appendix S1

## Data Availability

The data that support the findings of this study are available from Nevro Corp. Restrictions apply to the availability of these data, which were used under license for this study. Data are available from the author(s) with the permission of Nevro Corp.
