# Supplementary material for: Long‐term efficacy of 10 kHz spinal cord stimulation in managing painful diabetic neuropathy: A post‐study survey
Source: Pain Pract. 2025 Apr 17;25(5):e70023. doi: 10.1111/papr.70023 (PMC12004347; doi:10.1111/papr.70023)
Supplement: Supplementary file 1 — Appendix S1 [file PAPR-25-0-s001.docx]

Table S1. Comparison of last reported outcomes for patients who responded to the post-study survey versus those who did not. Analysis shows no significant differences between groups from pre-implantation baseline to last report.

| **Parameter** | **Post-study survey responders** | | **Analysis** |
| --- | --- | --- | --- |
|  | **Completers (N=57)** | **Non-completers (N=85)** |  |
|  | Mean Change (SD) | Mean Change (SD) | Difference (95% CI) |
| **Pain (VAS/NRS)** | -6.2(1.98) | -5.96 (2.39) | 0.241 (-0.489, 0.971) |
| **EQ5D5L Index** | 0.164 (0.159) | 0.135 (0.184) | -0.029 (-0.086, 0.028) |
| **Weight** (kg) | -7.3 (20.4) | -6.5 (25.2) | 0.78 (-6.97, 8.53) |
| **HbA1c** (%) | -0.37 (1.08) | -0.16 (1.48) | 0.21 (-0.27, 0.68) |
|  | N Reporting (%) | N Reporting (%) |  |
| **PGIC** |  |  | Chi-square test showed no significant association (p=0.299) |
| A Great Deal Better | 24 (42%) | 31 (36%) |  |
| Better | 19 (33%) | 30 (35%) |  |
